# A Review of Biomechanics Analysis of the Umbilical–Placenta System With Regards to Diseases

**DOI:** 10.3389/fphys.2021.587635

**Published:** 2021-08-12

**Authors:** Shier Nee Saw, Yichen Dai, Choon Hwai Yap

**Affiliations:** ^1^Department of Biomedical Engineering, National University of Singapore, Singapore, Singapore; ^2^Department of Bioengineering, Imperial College London, London, United Kingdom

**Keywords:** biomechanics, placenta, umbilical cord, elastography, tissue mechanics, fluid mechanics

## Abstract

Placenta is an important organ that is crucial for both fetal and maternal health. Abnormalities of the placenta, such as during intrauterine growth restriction (IUGR) and pre-eclampsia (PE) are common, and an improved understanding of these diseases is needed to improve medical care. Biomechanics analysis of the placenta is an under-explored area of investigation, which has demonstrated usefulness in contributing to our understanding of the placenta physiology. In this review, we introduce fundamental biomechanics concepts and discuss the findings of biomechanical analysis of the placenta and umbilical cord, including both tissue biomechanics and biofluid mechanics. The biomechanics of placenta ultrasound elastography and its potential in improving clinical detection of placenta diseases are also discussed. Finally, potential future work is listed.

## Introduction

The placenta is a fascinating organ that supports fetal growth throughout pregnancy. A successful development of the placenta and its vasculature is important for both the health of the mother and fetus. Abnormal placentation will result in diseases such as intrauterine growth restriction (IUGR) and/or pre-eclampsia (PE). IUGR and PE affect 5–10% of all pregnancy worldwide (Barut et al., 2010; Al-Jameil et al., 2014) and are the leading causes of fetal mortality and morbidity. IUGR is a pregnancy complication that the fetus did not receive sufficient nutrients due to placenta dysfunction. The adverse effects caused by placenta dysfunction toward fetus health do not end at the prenatal stage but continue to their adulthood life. For example, epidemiological studies showed that there is a significant association between IUGR and adult health outcomes such as diabetes and cardiovascular disease (Barker, 2006), suggesting that impaired prenatal development might lead to developing a wide range of adulthood diseases (Cheong et al., 2016). PE is a pregnancy complication where the mother experiences high blood pressure during pregnancy, which will affect placenta vasculature development. PE affects not only the fetus, but it is also detrimental to maternal health. It was reported that PE accounts for more than 50,000 maternal death worldwide every year (Ghulmiyyah and Sibai, 2012). Thus, it is pivotal for a successful placental development to ensure the lifelong health of both the fetus and mother.

Despite this important role of the placenta, our understanding of the placenta is limited, and this hinders our ability to invent novel and effective diagnosis and treatment tools for these pregnancy diseases. To this day, we have limited tools for treating or preventing placenta diseases. For this reason, there have been recent initiatives to intensify research on this organ. For example, Eunice Kennedy Shriver National Institute of Child Health and Human Development (NICHD) of the National Institutes of Health (NIH) initiated the *Human Placenta Project* in 2014, to decipher the function of the normal and pathological placenta (Guttmacher et al., 2014; Sadovsky et al., 2014) with the ultimate goal of improving disease detection and devising improved techniques to real-time monitor placenta function during pregnancy. Shortly after, China embarked on their own Human Placenta Project in 2015 to complement efforts in the US (Zhong and Zhong, 2016), and Europe embarked on the multicenter “iPlacenta” project in 2018. These growing interest and support are much welcomed by the research community.

To date, however, numerous studies about placenta have been focusing on the imaging and biological aspects. Placenta studies from the biomechanics point of view are not commonly adopted. Recent work has shown that the placenta and umbilical cord undergo biomechanical changes during disease, which could potentially be used as an evaluation parameter for disease detection. For example, the *in vivo* evaluation of the placenta stiffness via ultrasound elastography was suggested as a means to detect PE (Cimsit et al., 2015a,b; Kiliç et al., 2015; Alan et al., 2016b; Erbil et al., 2016) and IUGR (Quibel et al., 2015; Durhan et al., 2017; Habibi et al., 2017). Further, biofluid mechanics analysis of the placenta and evaluation of the umbilical-placenta vascular mechanical properties will improve our understanding about the placenta biotransport phenomenon and its vascular growth and remodeling process, respectively. The structure of this review first starts with basic biomechanics backgrounds, followed by the search methodology, the findings of the umbilical cord and placenta. Then, we introduce a non-invasive imaging tool in detecting pathological placenta—ultrasound elastography, by discussing its potential and its limitations and how biomechanics studies can improve this technology. Lastly, we conclude the review by discussing potential future research.

## Biomechanics Background

Biomechanics entails studies of the forces, movements, deformations and other physics of biological tissues. It can be broadly divided into biosolid mechanics (mechanics studies of tissue and structures) and biofluid mechanics (mechanics studies of fluids). Such study has made substantial contributions to our understanding of normal physiological functions and human diseases (Lee and Lim, 2007) where it reveals that biological tissues have the ability to sense environmental cues such as forces and deformations and respond to promote homeostasis in order to maintain tissue health (Hoefer et al., 2013; Humphrey et al., 2014; Baeyens et al., 2016). Some basic concepts involved are reviewed here.

### Biofluid Mechanics

In biofluid mechanics, the governing equations of fluid motion have been formulated as the Navier-Stokes equations, which consists of the continuity equation (Equation 1) that enforces the principle of conservation of mass, and the momentum equation (Equation 2) that enforces Newton's second law (that describes the relationship between forces and fluid acceleration).

(1) $$\nabla \cdot \vec{\text{U}}=0$$

(2) $$\frac{\partial \vec{\text{U}}}{\partial \text{t}}+\left(\vec{\text{U}}\cdot \nabla \right)\vec{\text{U}}=\;-\frac{1}{\rho }\nabla \;\text{p}+\;\text{v}\left(\nabla^{2}\vec{\text{U}}\right)$$

where ∇ is divergence, *t* is time, $\vec{U}$ is fluid velocity, *p* is fluid pressure, ρ is fluid density and *v* is kinematic viscosity.

For incompressible Newtonian fluids flow in a long straight tube under steady laminar condition, the Navier-Stokes equation can be simplified into the Hagen-Poiseuille flow, as stated in Equation 3.

(3) ΔP=QR

(4) $$\text{Q}=\;\pi {\text{r}}^{2}\cdot \frac{{\text{U}}_{\max}}{2}$$

(5) $$\text{R}=\frac{8\text{μ}\text{l}}{\pi {\text{r}}^{4}}$$

where Δ*P* is pressure drop, *Q* is flow, *R* is flow resistance, *r* is vessel's radius, *U*_*max*_ is maximum velocity of the flow profile, μ is dynamic viscosity and *l* is vessel's length.

**Figure 1:**
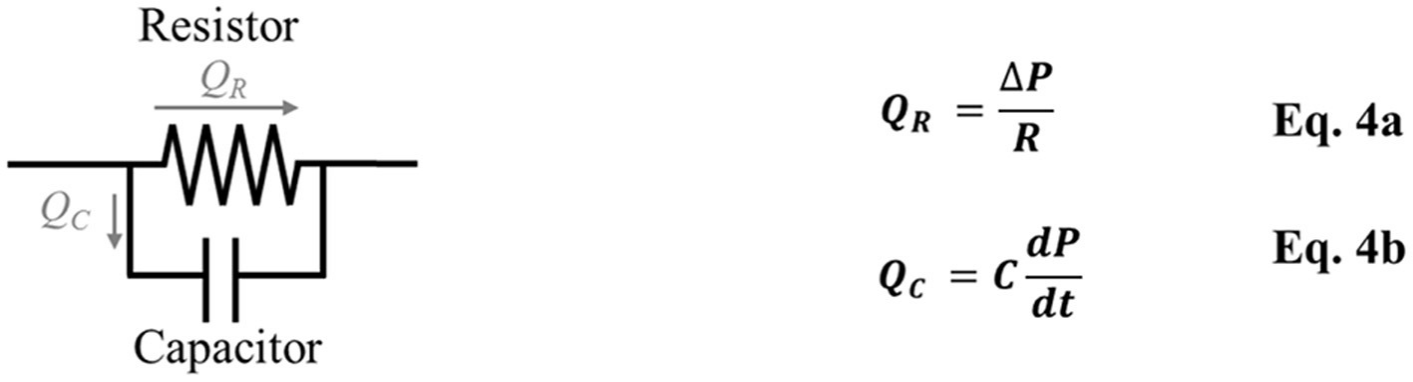
Windkessel model/Lumped-parameter modeling consists of a resistor, which represents vascular resistance (R), and a capacitor, which represents vascular compliance (C). *Q*_*R*_ is the blood flow through the resistor, *Q*_*C*_ is the blood flow through the capacitor, Δ*P* is the pressure difference, *dP/dt* is the pressure change over time.

In Equation 3, the pressure gradient acts as the driving force while the resistance acts as the retardation, and the interaction between these two factors determines the flow rate. This equation is equivalent to Ohm's law used in electrical circuits, where the potential gradient and electrical resistance determine the electric current, which is the flow rate of electrons. As such, investigators have used the electrical circuit to model blood flow in vascular networks such as that in the placenta (Reuwer et al., 1986; Thompson and Trudinger, 1990; Kleiner-Assaf et al., 1999), or to model boundary conditions of computational flow simulation (Laganà et al., 2005; Migliavacca et al., 2006). Such an approach is known as lumped-parameter modeling or Winkessel modeling (Figure 1) which consists of a resistor representing the vascular resistance and a capacitor representing the vascular compliance (Westerhof et al., 2009).

As blood fluid moves through the vessels or the heart, they interact with each other and with vascular walls, via frictional shear forces. It is crucial to quantify frictional shear forces because the endothelial cells are mechanosensitive. Frictional shear forces or wall shear stress (WSS) refers to the amount of shear force per unit surface area acting on the wall and can be expressed in the simplified form as shown in Equation 5.

(6) $$\tau =\;-\mu \frac{\partial \text{U}}{\partial \text{r}}$$

where μ is fluid dynamic viscosity and ∂*U/*∂*r* is velocity gradient near to the wall, in the axis that is perpendicular to the wall.

To date, the Navier-Stokes equations remain difficult to solve theoretically, and as such computational fluid dynamics (CFD) simulations are often performed to reveal flow patterns and forces in the cardiovascular system. CFD uses finite element approach and involves dividing the fluid body of interest into many small pieces or elements, and then uses computational means to iteratively calculate pressures and velocities at every node that will ensure that the Navier-Stokes governing equations are obeyed in every element. With CFD, we can gain insights into flow patterns that are difficult, expensive and even impossible to study using experimental techniques. Further, it can easily yield high spatial and temporal resolutions, which could be a challenge in experiments. It could also be used to predict flow phenomena in unencountered scenarios. To date, after numerous studies validating CFD simulations with experimental measurements (Armenante et al., 1997; Sheng et al., 1998; Montante et al., 2001), the confidence for its results are typically high. Past investigators have used CFD to understand the biomechanical behavior in the cardiovascular system (Steinman et al., 2003) and the placenta circulation system (Kaplan et al., 2010; Bappoo et al., 2017).

### Biosolid Mechanics

In biosolid mechanics, one major goal is to characterize the mechanical properties of tissues, so as to predict their deformational behavior under stress. As mechanical properties can be altered during disease, *in vivo* measurements of tissue stiffness can assist in diagnosis. In the simplest terms, mechanical properties of tissues can be described by the Hooke's Law, as shown below.

(7) $$\text{E}\;=\;\frac{\sigma (\text{ε})}{\text{ε}}$$

where σ is stress, ε is deformational strain and E is Young Modulus. In the soft biological sample, however, deformations are usually large and the stress-strain relationship is non-linear, and Equation 6 cannot accurately model it. Consequently, strain energy functions (SEF) have been utilized for this purpose, as they can properly model the 3-dimensional stiffness characteristics of tissues in a non-linear manner. The fundamental equation is shown in Equation 7, where the derivative of the SEF with respect to tissue deformation equals to stress (Fung, 2013).

(8) $$\sigma =\;\frac{\partial \text{W}}{\partial \text{ε}}$$

where σ is stress, ε is deformational strain and *W* is strain energy function. Various forms of SEFs have been proposed to describe the hyperelastic properties of different biological soft tissues such as breast, liver, and arteries (Samani and Plewes, 2004; Zulliger et al., 2004; Gasser et al., 2006; Chui et al., 2007). Simple SEF models like Neo-Hookean, Mooney-Rivlin, Odgen, and Yeoh models characterize isotropic material behaviors (Wex et al., 2015). More complicated models have been developed to cater for anisotropic material behaviors (Chui et al., 2007; Cai et al., 2016), such as the Holzapfel–Gasser–Ogden (HGO) model which incorporates collagen fiber orientation and reinforcement (Zulliger et al., 2004; Gasser et al., 2006). Moreover, generalized models that account for viscoelastic properties have been proposed (Miller and Chinzei, 2002; Panda and Buist, 2018).

In terms of blood vessels, their mechanical properties can be described in the simplified form, such as vascular compliance, *C*, which describes the vascular volume (*V*) changes with luminal pressure (*P*), such as in Equation 8, or they could be described using more complex SEF (Nolan et al., 2014).

(9) $$\text{C}=\;\frac{\text{Δ}\text{V}}{\text{Δ}\text{P}}$$

It is important to understand that biological tissue is usually complex, exhibits viscoelasticity properties with non-linear stiffness, where the stiffness could change with applied stress or strain, and anisotropy. Thus, there are substantial challenges in providing very accurate models. However, models with some simplifications have often been found to be very useful.

## Search Methodology

We used Google Scholar and PUBMED database and search for keywords “placenta,” “umbilical cord,” “umbilical Doppler,” “biomechanics,” “fluid dynamics,” “tissue mechanics,” “finite element,” “computational fluid dynamics,” “Wharton Jelly,” “stiffness,” “IUGR,” “growth restriction,” “preeclampsia,” “strain elastography,” “shear wave elastography,” and “acoustic radiation force impulse.” Combinations of the keywords with AND/OR Boolean operators were used. We also searched through the “related articles” and “cited by” articles to widen the search space. No publication year limit was applied. We included articles of human and animal biomechanical studies; for human studies, all maternal age-range of population was included. For elastography review, we only included studies related to human. We included journal, conference and book publications, but did not include theses and non-English articles. However, we included only top search results that are highly representative and relevant, and did not include publications with similar content and repeated conclusions, to avoid an overly lengthy manuscript.

## Umbilical Cord

### Umbilical Vessels

**Figure 2:**
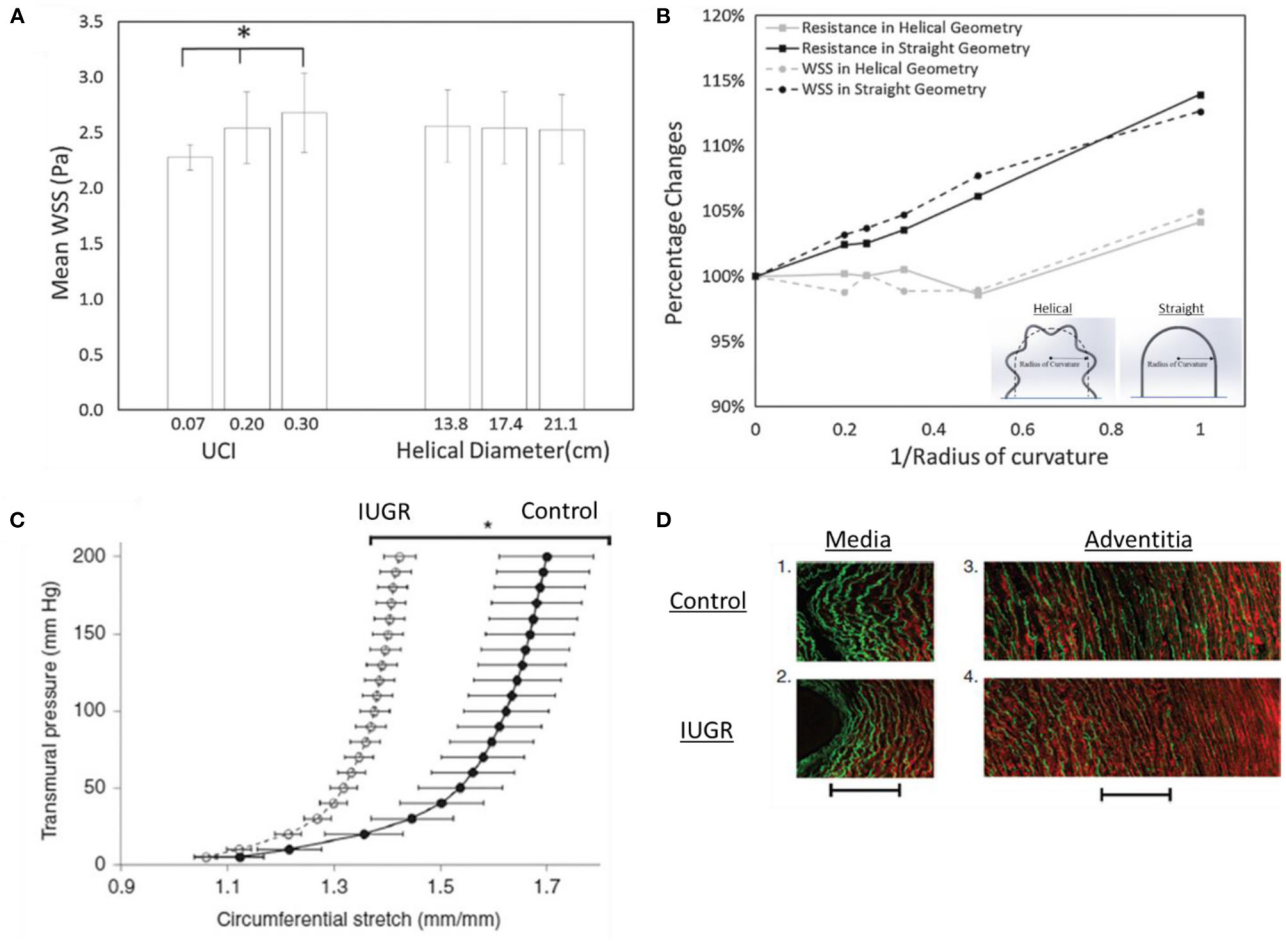
Biomechanics in umbilical arteries. **(A)** The flow resistance and wall shear stress (WSS) are affected by the umbilical coiling index (UCI) but not helical diameter. **(B)** Flow resistance and WSS disruption in helical geometry are lesser as compared to straight geometry, suggesting that the helical geometry of umbilical arteries aids in minimizing WSS disruption. This may indicate the existence of the WSS homeostasis mechanism in umbilical arteries for vascular growth (Saw et al., 2017). **(C,D)** IUGR umbilical arteries stretch lesser compared to control, indicating stiffer properties. The increased stiffness could be due to the increased collagen (red) and decreased elastin (green) contents shown by second-harmonic generation images (Dodson et al., 2013b). * indicates *p* < 0.0001.

The umbilical cord consists of two umbilical arteries and one umbilical vein. The umbilical artery has an interesting helical structure where it coils around the umbilical vein. These helical structures can be observed at the 7th weeks of post-conception (de Laat et al., 2005). Some authors hypothesized that the formation of helical structures could be due to the helical muscle layer in the umbilical cord (Spurway et al., 2012) and its helical pattern was affected by *in utero* fetal movements (Lacro et al., 1987). Biomechanics studies of umbilical blood vessels have suggested an interesting function for umbilical arterial helical structure. Finite Element (FE) simulation showed that helical structures of umbilical arteries aids in maintaining feto-maternal thermoregulation in the placenta circulation (Kasiteropoulou et al., 2020). Interestingly, experimental study showed that umbilical venous flow resistance did not increase up to 30–50% of compression of cord which could be due to the poroelastic behaviour of Wharton Jelly (Pennati et al., 2013). Using clinical ultrasound-images and CFD of umbilical vessels, Saw et al. showed that the helical shape of umbilical arteries protected it from flow conditions changes in the event of cord bending since arteries were already curved even before bending, but the same was not true of umbilical veins, as shown in Figure 2, (Saw et al., 2017). They further showed that umbilical arteries had consistent WSS, but not umbilical veins, suggesting that (1) the arteries maintained a WSS homeostasis via vascular growth and vascular tone regulation and (2) arteries were more responsive to WSS, and thus required a more consistent fluid mechanical environment. WSS homeostasis refers to the concept that blood vessels regulate their diameter to maintain a constant physiological flow shear stress level (Fung, 1990). Another CFD simulation showed that the large WSS difference between systole and diastole may contribute to the helical structures of umbilical arteries (Wen et al., 2020).

In the clinical setting, the coiling of the umbilical artery is represented by the umbilical coiling index (UCI) which describes the number of coils in one cm. A normal UCI is 0.2 (0.2 coils per cm) while hypercoiled and hypocoiled arteries are 0.3 (90th centile) and 0.03 (10th centile), respectively (Strong et al., 1994). It was shown that UCI was highly correlated with adverse perinatal outcomes (Chitra et al., 2012). For example, hypocoiled artery was associated with hypertension disorder, low birth weight, fetal heart rate abnormalities while hypercoiled artery was associated with diabetes mellitus and congenital abnormalities (Chitra et al., 2012). As for IUGR, both hypocoiling and hypercoiling were reported but hypercoiling is more commonly observed in IUGR (Devaru and Thusoo, 2012; Mittal et al., 2015; Chholak et al., 2017; Rahi and Akther, 2017). CFD study showed that UCI has a significant effect in umbilical artery blood flow hemodynamics, whereby a hypercoiled artery experienced greater pressure drop and WSS (and thus higher flow resistance) as compared to hypercoiled artery, as shown in Figure 2, (Kaplan et al., 2010; Saw et al., 2017; Shah et al., 2017). Studies also showed that the umbilical arteries can incur substantial pressure drop across its length (Kaplan et al., 2010), suggesting that with hypercoiled arteries, the fetal body could be prompted to adopt hypertension to ensure sufficient flow through the placenta. A recent study used computational modeling and proposed the Flow and Pressure Indices to estimate the umbilical cord flow resistance and pressure drop across the umbilical cord. The indices were computed from the cord length, cord width and the number of coiling and could be useful in clinical settings (Wilke et al., 2021).

Numerous studies have shown that during IUGR, the umbilical blood flow rate significantly decreases (Najafzadeh and Dickinson, 2012; Parra-Saavedra et al., 2013a). Some authors claimed that the reduction of venous flow was mainly due to the reduction of umbilical venous size (Bruch et al., 1997) while others claimed that it was due to the reduction of venous velocity (Ferrazzi et al., 2000; Rigano et al., 2001). There is also evidence that the umbilical vascular sizes (both arteries and vein) do not increase with gestational age in IUGR (Boito et al., 2002; Raio et al., 2003; Saw et al., 2018c), suggesting that the vessels are not growing in tandem with the fetal growth. Biomechanics approaches can again be useful in this area. It is known that in other blood vessels, mechanical forces such as vascular wall stresses and fluid flow WSS modulates vascular growth and remodeling (Hoefer et al., 2013; Humphrey et al., 2014), and the same could well be true for umbilical and placental vessels, which detailed review can be found in (Morley et al., 2019). For example, it was shown that WSS remained constant across gestational age and vascular diameter in human umbilical artery (Saw et al., 2018c), human fetal aorta (Struijk et al., 2005) and fetal mice aorta (Yap et al., 2014), suggesting that the blood vessels adapt to blood flow by regulating their diameter to promote WSS homeostasis so as to maintain vessels' health.

Biomechanics studies in normal and IUGR umbilical vessels showed that the WSS characteristics of these vessels remain unchanged (Saw et al., 2018c), thus suggesting that the reduced flow rates and oxygen contents were more likely the factors for the lack of growth in IUGR umbilical vessels. Further, studies on human umbilical arteries from IUGR pregnancy showed that these arteries had thinner vascular walls (Bruch et al., 1997) and exhibited greater stiffness (Burkhardt et al., 2009). The increased stiffness of the IUGR umbilical artery was accompanied by a reduction in insulin-like growth factor-I (IGF-I), which was responsible for elastin synthesis (Burkhardt et al., 2009), and such a change in vascular fiber structure can lead to changes in vascular mechanical properties. Similar observation was obtained in IUGR sheep, as shown in Figure 2, (Dodson et al., 2013b) as well as PE in humans (Pawlicka et al., 1999; Dodson et al., 2013a; Herzog et al., 2017) whereby the stiffness of umbilical arteries was greater in disease groups. However, opposite observation was reported in IUGR guinea pig's umbilical arteries in which the stiffness showed no significant difference between control and disease (Cañas et al., 2017). These arteries had increased collagen-to-elastin ratio which contributed to anisotropic stiffening and increased sulfated glycosaminoglycans content (GAGs) which contributed to isotropic stiffening. Dodson et al. (2013a) also reported a significant increase in pre-eclamptic circumferential elastic modulus which corroborated with higher pulsatility index (PI) observed in PE. Collectively, it is believed that the vascular remodeling process in IUGR and PE umbilical arteries could have been altered and the material properties changes might contribute to the abnormal umbilical artery Doppler flow observed in IUGR and PE.

### Wharton's Jelly

**Figure 3:**
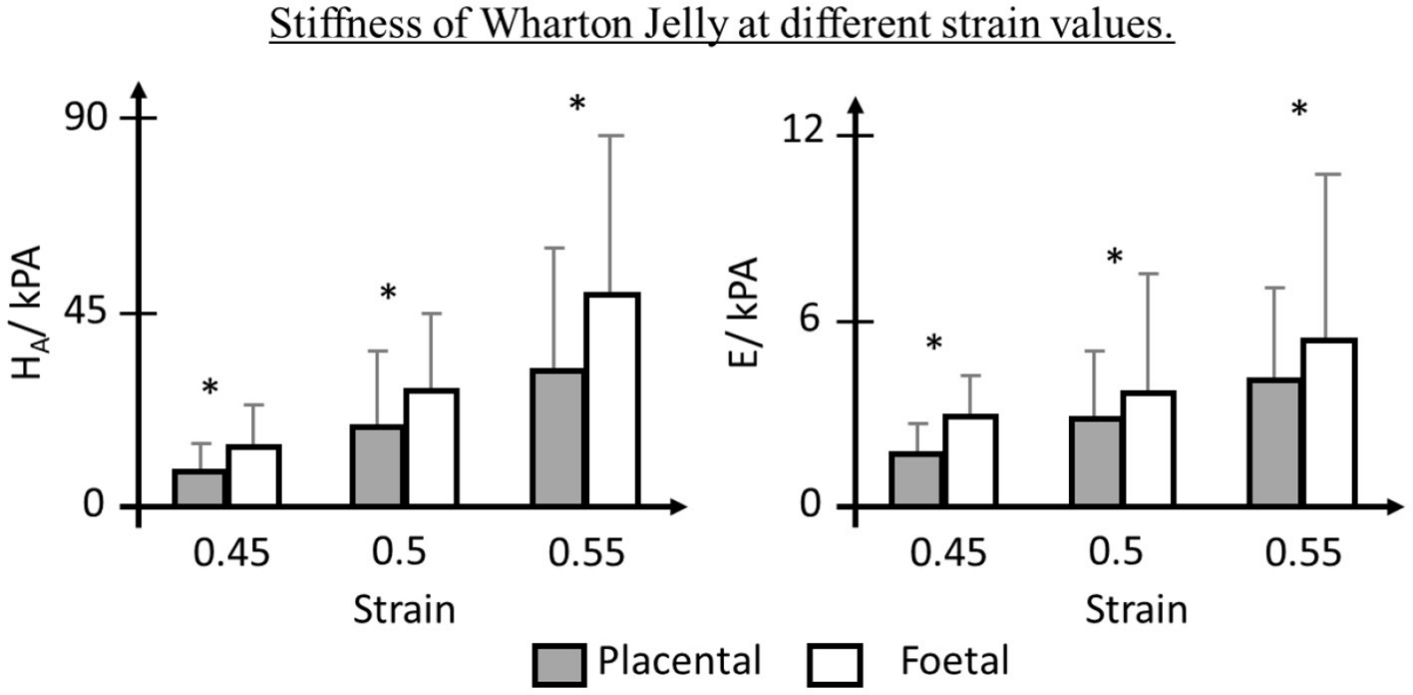
Biomechanics of Wharton Jelly (WJ). WJ near placenta insertion showed lower Aggregate Modulus (H_A_) and Young Modulus (E), depicting non-homogeneous mechanical properties (Gervaso et al., 2014). * indicates *p* < 0.05.

Surrounding the umbilical vessels is the WJ, a spongy structure with pores filled with hyaluronic acid (HA) and proteoglycans, hydrophilic molecules that resist compression (Ferguson and Dodson, 2009; Davies et al., 2017). It has been suggested that the WJ plays an important role in vascular support and umbilical blood flow regulation (Pennati, 2001; Ferguson and Dodson, 2009; Gervaso et al., 2014; Davies et al., 2017; Brunelli et al., 2019). The WJ was reported to share similar tensile and viscoelastic behaviors with longitudinal segments of the umbilical vein (Pennati, 2001). It displayed non-linear stress-strain behavior as well as elasticity due to the interlacing network of collagen fibers and glycoprotein microfibrils, respectively. Compliance tests were conducted on umbilical veins with and without WJ, respectively and it was found that umbilical veins with the WJ were significantly stiffer, suggesting the great influence of WJ to circumferential strength of the umbilical cord. Significantly different compressive poroelastic parameters of the perifetal and periplacental WJ were reported, showing non-homogeneous mechanical properties of the WJ (Figure 3) (Gervaso et al., 2014). Brunelli et al. (2019) then reported a remarkably stiffer WJ from the fetal side through planar equibiaxial tension tests. They also used finite element modeling to simulate stress distribution in the umbilical cord and found that WJ assisted in stress transmission and redistribution, thus regulating umbilical cord blood flow. A smaller WJ area was reported in IUGR (Raio et al., 2003) or PE (Raio et al., 2002; Romanowicz and Galewska, 2011; Herzog et al., 2017) and this could increase the risk of umbilical cord compression (Silver et al., 1987; Brunelli et al., 2019). Pre-eclamptic WJ was also found to have premature replacement of HA by sulfated GAGs and increased collagen content which dehydrate the umbilical cord and decrease the elasticity (Bańkowski et al., 1996; Pawlicka et al., 1999). Therefore, the alterations of WJ in IUGR and PE can be the contributing factors for the altered umbilical cord hemodynamics in these fetuses.

## Placenta

### Placenta Blood Flow Modelling

The placenta is a transient organ that forms to supply oxygen and nutrients to the fetus during pregnancy. In order to ensure sufficient blood flow in the placenta, the development of the placenta vasculature network is very crucial.

Researchers have shown that vascular corrosion casting and imaging (MRI, micro-CT scan, or scanning electron microscopy) techniques are effective in studying the placenta vasculature network morphology (Habashi et al., 1983; Leiser et al., 1997; Rennie et al., 2017; Link et al., 2020). A more detailed of 3D placenta imaging is reviewed in Lewis and Pearson-Farr (2020). From vascular corrosion casting studies, categorization into two types of chorionic arterial branching patterns was proposed, which were the dichotomous and monopodial branches (de los Vasos Sanguíneos, 2012). In dichotomous branches, the two daughter vessels were similar in their sizes, while in monopodial branches, one daughter vessel was much larger than the other. Investigators have proposed that dichotomous branches are more suitable for distributive flow to the local region, while monopodial branches are more suitable for transporting blood to locations further from the umbilical cord (Gordon et al., 2007b). This corroborated with observations that the vasculature network was mostly monopodial for placentas with marginal cord insertion, and dichotomous for placentas with central cord insertion (Gordon et al., 2007a).

Corrosion casting studies revealed that IUGR had significantly decreased placenta volume (Link et al., 2020), vascular volume (Langheinrich et al., 2008), lumen size (Junaid et al., 2014), arterial length (Junaid et al., 2017), and increased venous length (Junaid et al., 2017). The morphological changes observed in casting studies corroborated with histological evidence where the surface area and volume of intervillous space and villi were significantly reduced in IUGR (Mayhew et al., 2003, 2007). Similar findings were also observed in PE placenta where the chorionic arteries diameter in PE placenta were smaller (Yin et al., 2017) but the volume of intervillous space and villi diameter was similar to that of in normal placenta (Mayhew et al., 2003). In other studies, interestingly, the branching angle was shown to have a negative association with babies' birth weight (Haeussner et al., 2014) and that branching angle of placental terminal villi in IUGR was larger than that of in normal (Haeussner et al., 2016). In a similar study, it was reported that IUGR has smaller tortuosity at preterminal villi, Haeussner et al. suggested that the morphological changes in IUGR placenta could be an adaption toward insufficient uterine spiral arterial remodeling (Haeussner et al., 2016).

**Figure 4:**
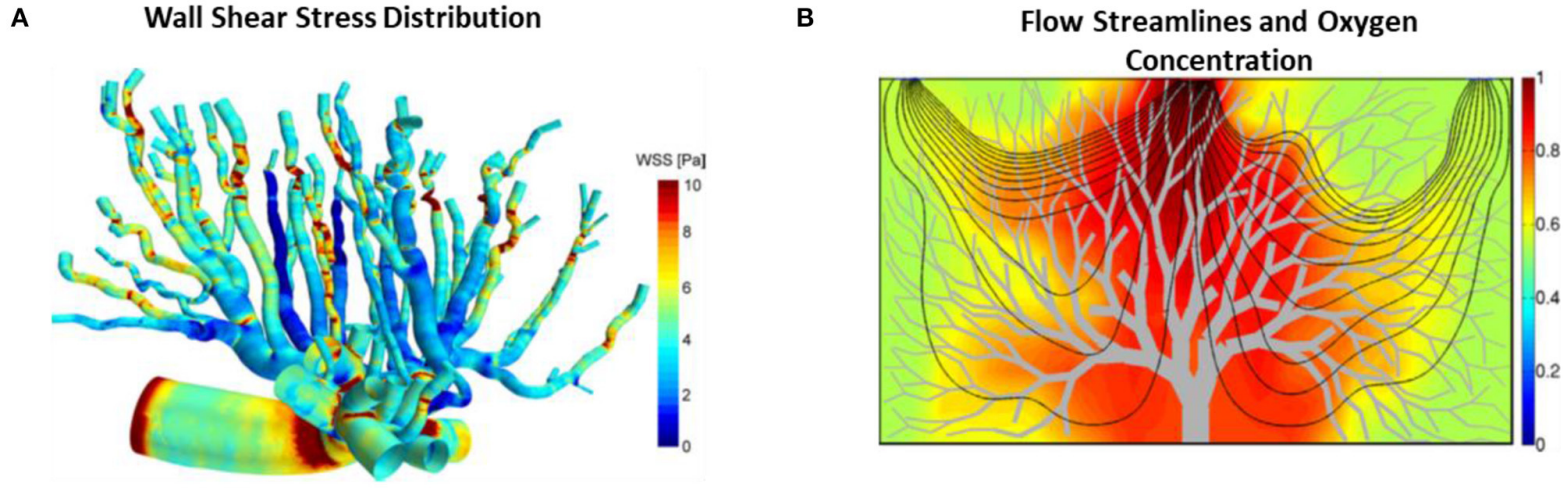
Placental blood flow modeling. **(A)** WSS color contours on mouse placenta vasculature performed using CFD (Bappoo et al., 2017). **(B)** Blood flow streamlines and oxygen concentration field in the 2D placenta model (Lin et al., 2016).

To understand the hemodynamics and blood transport phenomena in the placenta, biomechanical computational modeling of network blood flow had been performed (Clark et al., 2015, 2021; Junaid et al., 2017; Bappoo et al., 2021). One such multiscale modeling of placenta vasculature showed that the location of cord insertion and placenta shape with similar volume did not affect the overall placenta flow resistance, rather, the major determinants of placenta resistance were vessel sizes and numbers (Clark et al., 2015). In another study (Figure 4), it was reported that the oxygen exchange was sensitive to villous length whereby an increase of villous branch length reduced the oxygen uptake rate (Lin et al., 2016). Their results could explain the increased venous length observed in IUGR from Junaid et al. (2017). This is because oxygenated blood is mainly carried by venous in the placenta and the increased venous length would result in reduced oxygen uptake rate and thus affect fetal growth. As such, our understanding on the fetal-maternal circulation is important. Computational modeling can be a valueble tool to enhance our understanding normal and pathological placenta circulation. Bappoo et al. recently proposed a robust framework for hemodynamics analysis in placental circulation (Bappoo et al., 2021). A comprehensive review on such modeling is provided by Clark et al. (Clark et al., 2021).

It is important to understand the biomechanical environment in placenta because fetoplacental vessels have no nervous innervation (Myatt, 1992) and thus some authors have suggested that these vessels rely on mechanical stresses stimuli for their vasotone control and growth and remodeling, given that there is strong evidence in other blood vessels for such a mechanism (Lu and Kassab, 2011; Hoefer et al., 2013; Matsumoto et al., 2015; Baeyens et al., 2016). CFD simulation was recently performed on mouse placenta vasculature (Bappoo et al., 2017; Shannon and Mirbod, 2017) to understand the *in vivo* mechanical environment and fluid patterns in the placenta (Figure 4). In a recent report, WSS gradient was found to be larger at a bifurcation as compared to neighbor vessels, and the authors suggested that this may induce the enlargement, sprouting and pruning of vessels during angiogenesis (Bappoo et al., 2017), which was corroborated with Dolan et al. study whereby they showed that the positive WSS gradient favored vascular proliferation (Dolan et al., 2013). Furthermore, the production of nitric oxide (NO) was affected by WSS in human fetoplacental arteries (Wieczorek et al., 1995; Li et al., 2003; Sprague et al., 2010). NO is the most prominent vasodilator produced during pregnancy that plays a critical role in vasculogenesis and angiogenesis (Zullino et al., 2018). *In vitro* experiment showed that human placenta arteries were sensitive to NO, for example, it was shown that the placental arteries dilated less in the presence of NO synthase inhibitor and more in the absence of NO synthase inhibitor (Learmont and Poston, 1996). In another *in vitro* study, endothelial cells were observed to migrate slower in high shear stress biomechanical environments, which is likely to hamper vascular development (Tun et al., 2019). This corroborates with the findings that severe IUGR placenta microvascular vessels experience higher WSS (Tun et al., 2019). Another study by Martin et al. observed enhanced NO production in fetoplacental microcirculation with pulsatile flow condition than that of in non-pulsatile flow condition, suggesting pulsatile flow property may affect vasomediation process (Martin et al., 2020). Further, animal experiments showed that inhibition of NO production during pregnancy results in the impoverished placenta vascularization (Tarrade et al., 2014), and induced placental insufficiency (Yallampalli and Garfield, 1993; Edwards et al., 1996). As such, gaining understanding about the biomechanical environment in the placenta could shed some light on the production of NO and hence the placental vascular growth and remodeling process during pregnancy.

The flows in the umbilical and placental arteries are pulsatile and are often evaluated via umbilical artery Doppler indices (Equation 9) such as Resistance Index (RI) or Pulsatility Index (PI). These indices are thought to be reflective of the flow conditions in the placenta, and are widely used in evaluating placenta function and have been used in clinics to monitor fetus health.

(10) $$Resistance\;Index\;\left(RI\right)=\;\frac{PSV-EDV}{PSV}$$

(11) $$Pulsatility\;Index\;\left(PI\right)=\;\frac{PSV-EDV}{TAV}$$

where PSV is peak systolic velocity, EDV is end diastolic velocity and TAV is time-averaged velocity. RI and PI were most directly thought of as indicators of the downstream placenta resistance, as strong evidence in the literature showed this relationship (Trudinger et al., 1987; Adamson et al., 1989; Krebs et al., 1996). As such, these indices are often used in clinics to evaluate the placenta function and differentiate between disease babies, who suffer from placental insufficiency, and small but healthy babies (Figueras and Gardosi, 2011). Krebs et al. study also showed direct evidence that an impoverished growth of placental vessels contributed to abnormal umbilical Doppler velocity flow (Krebs et al., 1996), further supported this notion.

The functional relationships between Doppler indices and placental vascular hemodynamics is largely a biomechanics problem and could be modeled. For example, Reuwer et al. used of electric circuit equivalent model/lump parameter model in the umbilical-placental circulation to analyze hemodynamics, and they found that the umbilical arterial PI was affected by fetal aortic pulse pressure and suggested to average several PI values over a period to diminish the impact of aortic pulse pressure on umbilical arterial PI (Reuwer et al., 1986). Subsequently, Thompson et al. developed another computational model for the umbilical-placental circulation in the pulsatile flow state and revealed that the relationship between umbilical PI was not linear with placenta resistance, for example, PI increased gradually at first and increased sharply after there was 60% placenta obliteration (Thompson and Trudinger, 1990). Furthermore, they showed that placenta with high resistance was able to maintain volumetric output when aortic pressure was increased (Thompson and Trudinger, 1990). Similar finding was observed in Surat et al. model whereby they showed that a 300% increased in placenta resistance was needed to observe a zero end-diastolic flow in the umbilical artery (Surat and Adamson, 1996). As for umbilical length, blood viscosity and mean arterial pressure, it was shown that these parameters had little effect on Doppler indices (van den Wijngaard et al., 2006). These studies thus showed that drastic changes would have occurred to the placenta vessels before abnormal Doppler indices present itself, and this index may not be useful for disease cases that are not as severe. In corroboration, investigators reported that some IUGR fetuses have normal Doppler indices even though their placenta depicted lesion and inflammation, displaying a sign of increased placenta resistance (Parra-Saavedra et al., 2013b). Moreover, a multicenter prospective PORTO study, in fact, reported that only 40% of IUGR fetuses showed abnormal umbilical arterial blood flow while the remaining 60% of IUGR have normal umbilical arterial blood flow (Unterscheider et al., 2013).

### Placenta Vascular Mechanics

Given that studies have found limits to how strongly umbilical Doppler Indices can correlate with placental vascular resistance, it is logical to consider that other factors may be affecting Doppler indices, and one such candidate is vascular compliance or the flexibility of the vessels. In fact, In an *in vitro* experiment involving a flow loop with flexible tubing, it was shown that the flow pulsatility and RI depended on both the resistance and compliance, instead of just resistance (Bude and Rubin, 1999). Biomechanical modeling work also provided some insight on this relationship between placenta vascular compliance and umbilical arterial blood flow waveforms, showing that Doppler indices varied inversely with placenta arterial elasticity (Surat and Adamson, 1996; Kleiner-Assaf et al., 1999; van den Wijngaard et al., 2006).

There is evidence that suggested that during IUGR disease, placenta vascular structure and stiffness changed. For example, in our recent experiment, we observed that in IUGR pregnancies with abnormal Doppler waveforms, the chorionic arteries displayed higher vascular compliance or reduced vascular wall stiffness (Saw et al., 2018d). Similar finding was also observed in patient-specific mathematical modeling study where it was shown that placental compliance was increased in IUGR and was positively associated with UA PI (Garcia-Canadilla et al., 2015). Wareing et al. also reported increased chorionic venous contractility induced by hypoxia in IUGR but not in normal pregnancy (Wareing et al., 2006). Furthermore, previous studies reported that placental terminal villi thickness was increased in mouse (Hvizdošová-Kleščová et al., 2013) and in human placenta from IUGR pregnancy (Mayhew et al., 2003), and elastic fibers in IUGR placenta blood vessels were found to be reduced (Wilhelm et al., 2002) but increased in PE pregnancy (Baran et al., 2010). Collectively, their results suggested that the vascular stiffness/compliance might have altered in disease and it could affect the blood flow hemodynamics in fetoplacental circulation. These results suggested that aside from placenta resistance, placenta compliance should also be taken seriously when studying fetoplacental hemodynamics and indices used clinically, and biomechanics can provide excellent tools for such studies.

### Placenta Mechanical Properties

The mechanical properties of the human placenta were first investigated with the purpose of investigating the effect of car crashes on the placenta so as to improve the safety features in car design (Manoogian et al., 2008, 2009; Delotte et al., 2009; Hu et al., 2009, 2011; Weed et al., 2012; Pérès et al., 2014). In their studies, it was shown that the placenta exhibited typical non-linear stress-strain relationship (Pérès et al., 2014) and had viscoelastic properties whereby its' mechanical behavior changes with strain rate (Manoogian et al., 2009). Investigations of placenta mechanical properties are useful because their outcomes can be used for computational modelling of normal or diseased clinical events or during accidents to predict risks and outcomes. For example, a recent study developed a FE model of the uterus, placenta, fetus and amniotic fluid, and investigated the biomechanical effects of a blunt trauma on the pregnant abdomen (Irannejad Parizi et al., 2020). A hyperelastic model such as the Ogden model was commonly used in the past to describe the mechanical properties of the placenta during loading conditions (Hu et al., 2009; Pérès et al., 2014; Saw et al., 2018b). Recently, models were developed by taking the viscoelastic properties of the placenta into account and such models were able to describe the stress-strain relationship of the placenta during loading and unloading conditions well (Lau et al., 2016; Panda and Buist, 2018).

Histological studies had shown abundant shreds of evidence that the diseased placenta had considerably alteration in placental microstructures in which diseased placenta had increased syncytiotrophoblast apoptosis, fibrosis (Allaire et al., 2000; Roberts and Post, 2008; Scifres and Nelson, 2009; Güven et al., 2018), thicker trophoblast epithelium (Langheinrich et al., 2008), reduced elastin, and increased collagen (Macara et al., 1996; Wilhelm et al., 2002), suggesting that the mechanical properties of the placenta during disease could have been altered but no direct proof about the placenta mechanical properties was shown. Direct mechanical testing of normal and IUGR placenta was recently conducted and the mechanical properties of IUGR placenta were found to be stiffer than that of the normal placenta and the difference could be captured from constitutive models' parameters (Saw et al., 2018b). However, the placenta stiffness difference was only significant at certain compression settings, suggesting that the use of non-invasive ultrasound elastography could be used in detecting disease placenta but the setting of placental elastography should be controlled wisely to enhance its' accuracy.

*In vitro* experiments had shown that hypoxic condition was shown to lead to increased production of collagen (Agocha et al., 1997; Chen and Aplin, 2003; Kowalski et al., 2015). IUGR placenta, in fact, was found to have a greater amount of collagen to elastin ratio as compared to that of in normal (Saw et al., 2018b), which corroborated with the *in vitro* experiment results (Agocha et al., 1997; Chen and Aplin, 2003; Kowalski et al., 2015). The increased amount of collagen in IUGR suggested that the IUGR placenta was in hypoxic condition and thus increased in its stiffness.

## Placenta Ultrasound Elastography

**Figure 5:**
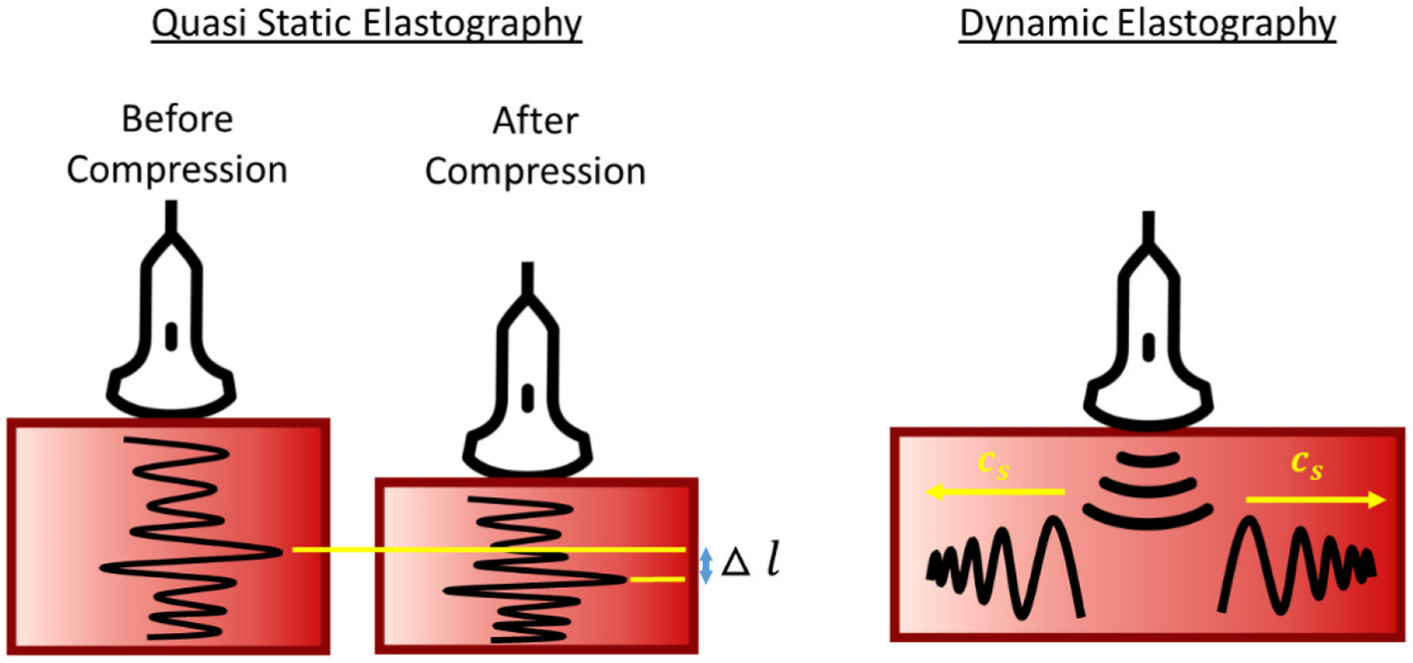
Schematic diagram of quasi-static and dynamics elastography. Stiffness is represented by tissue deformation and propagation wave speed in quasi-static and dynamic elastography, respectively.

Ultrasound elastography, an emerging imaging tool to measure tissue stiffness non-invasively (Ophir et al., 2002), has been widely used in the clinic for breast tumors and liver cirrhosis diagnosis (Krouskop et al., 1998; Ferraioli et al., 2014). Recently, elastography has been extended to evaluate the placenta and has shown promise to improve the detection of placenta abnormalities such as IUGR and PE (Table 1). As Table 1 shows, multiple investigations, including both *in vivo* and *ex vivo* ones, demonstrated that elastography can measure appreciable differences between normal and diseased. Elastography essentially makes measurements of tissue behavior that reflect its mechanical properties, and if tissue mechanical properties change during disease, elastography can be used to detect such changes to detect the disease. There are two types of ultrasound elastography, namely quasi-static elastography/ strain imaging (SE), and dynamic elastography/ shear wave imaging (SWI). A schematic diagram of the quasi-static and dynamic elastography is illustrated in Figure 5.

**Table 1:** Elastography measurements on human placenta.

		**Normal**		**IUGR**		**PE**	
**SE**	**Scan GA**	***n***	**Elasticity**	***n***	**Elasticity**	***n***	**Elasticity**
***Ex vivo***							
Durhan et al. (2017)	35–42nd week	30	EI: 0.80 ± 0.22	25	EI: 0.58 ± 0.21^*^	–	–
^§^Saw et al. (2018a)	38–41st week	6	PPSR: 2.4 (1.5–3.3)	3	PPSR: 2.5 (2.2–3.1)		
***In vivo***							
^§^Cimsit et al. (2015b)	20–23rd week	101	EI: 0.9 (0.82–0.97)	–	–	29	EI: 1.56 (1.12–2.16)^*^
^§^Albayrak et al. (2016)	28–42th week	70	FPSR: 5.0 (0.2–14.0)	–	–	–	–
**ARFI**	**Scan GA**	***n***	**Elasticity**	***n***	**Elasticity**	***n***	**Elasticity**
***Ex vivo***							
Sugitani et al. (2013)	26–41th week	115	SWS: 1.31 ± 0.35 m/s	24	SWS: 1.94 ± 0.74 m/s^*^	–	–
McAleavey et al. (2016)	Not stated	11	YM: 1.92 ± 0.05 kPa SWS: 0.8 ± 0.13 m/s^b^	–	–	–	–
***In vivo***							
Ohmaru et al. (2015)	17–40th week	143	SWS: 0.98 ± 0.21 m/s	21	SWS: 1.28 ± 0.39 m/s^*^	–	–
Alan et al. (2016a)	18–28th week	34	SWS: 1.09 ± 0.20 m/s	–	–	–	–
Wu et al. (2016)	2nd−3rd trimester	100	SWS: 0.98 ± 0.25 m/s	–	–	–	–
^†^Alan et al. (2016b)	2nd−3rd trimester	44	SWS: 1.07 (1.00–1.14) m/s	–	–	42	SWS: 1.39 (1.32–1.53) m/s^*^
Erbil et al. (2016)	3rd trimester	38	SWS: 0.91 ± 0.20 m/s	–	–	35	SWS: 1.93 ± 0.62 m/s^*^
^§^Cimsit et al. (2015a)	20–23th week	101	YM: 2.53kPa (2.29–2.80) SWS: 0.91 m/s (0.87–0.97)^b^	–	–	28	YM: 7.0kPa (3.79–13.3)^*^ SWS: 1.53 m/s (1.12–2.11)^b^
Akbas et al. (2019)	29-37th week	81	YM: 3.85 ± 1.2 kPa	66	YM: 5.51 ± 2.09 kPa^*^	–	–
Hefeda and Zakaria (2020)	2nd trimester	45	SWS: 0.85 ± 0.43m/s	–	–	9	SWS: 2.13 ± 1.48 m/s
Hefeda and Zakaria (2020)	3rd trimester	94	SWS: 0.89 ± 0.57m/s	–	–	46	SWS: 2.23 ± 1.48 m/s
**SWE**	**Scan GA**	***n***	**Elasticity**	***n***	**Elasticity**	***n***	**Elasticity**
***In vivo***							
^¥^Kiliç et al. (2015)	2nd−3rd trimester	27	YM: 4.00 (1.5–14) kPa	–	–	23	YM: 21.0 (3–71) kPa^*^
Yuksel et al. (2016)	Median = 30.5th week	43	YM: 5.47 ± 1.17 kPa	–	–	–	–
^†^Habibi et al. (2017)	2nd−3rd trimester	42	Maternal side: YM: 6.0 (4.38–7.45) kPa Fetal side: YM: 5.0 (3.73–6.55) kPa	42	Maternal side: YM: 28 (16.8–35.0) kPa^*^ Fetal side: YM: 21.5 (13.5–28.0) kPa^*^	–	–
Spiliopoulos et al. (2020)	2nd trimester	6	YM: 18.16 ± 11.6 kPa	–	–	5	YM: 27.23 ± 10.1 kPa
	3rd trimester	18	YM: 10.45 ± 7.6 kPa	–	–	18	YM: 26.36 ± 14.1 kPa^*^
Abeysekera et al. (2017)	37–41th week	61	YM: 10.8 (5–17) kPa	–	–	–	–

*Data shown in mean ± SD unless stated otherwise. GA, Gestational Age; PPSR, Placenta-to-PVA Strain Ratio; FPSR, Fat-to-placenta Strain Ratio; ARFI, Acoustic Radiation Force Impulse; SWS, Shear Wave Speed; SWE, Shear Wave Elastography; YM, Young Modulus; SE, Strain Elastography; IUGR, Intrauterine Growth Restriction; PE, Pre-eclampsia; n, sample size*.

† *data shown in median (interquartile range);*

¥ *data shown in median (range)*.

§ *data shown in mean (range)*.

* *p < 0.05*.

b *SWS was computed using Equation 10*.

During SE, operators perform manual palpation on the tissue and SE measures tissue deformations during the palpation as a proxy for tissue stiffness. The stiffness of a targeted tissue is expressed as strain ratio, the strain of targeted tissue to the strain of reference tissue. In several implementations, maternal fat tissues were used as the reference tissue (Cimsit et al., 2015b; Albayrak et al., 2016). A major difficulty, however, is reproducibility. Firstly, different machines have different definition of elasticity index (EI) or strain ratio, for example, Hitachi expressed EI as strain experienced by tissue during displacement, thus higher EI indicates softer tissue (Durhan et al., 2017) while GE machine expressed EI as stress needed to compress the tissue, thus, higher EI indicates stiffer tissue (Cimsit et al., 2015b). Furthermore, it is difficult to maintain constant palpation among operators, and readings between patients can be difficult to compare. In Albayrak et al. study, fat-to-placenta strain ratio was reported from SE instead and showed a large variability in his results, range from 0.2 to 14.0 for normal placenta (Albayrak et al., 2016). On top of palpation inconsistency, this large variability of the readings could also be due to person-to-person variability of fat tissue stiffness, which acted as the reference tissue. In fact, studies showed that fat tissue stiffness can vary substantially from one person to the next and have large standard deviations (Alkhouli et al., 2013). In order to maintain a consistent compression setting, it was recently proposed that SE palpation should be conducted via motorized motion and that an external PVA gel as reference tissue, which showed appreciable improvement to measurement variability (Saw et al., 2018a). However, despite imperfect precision, investigators have nonetheless found SE measurements capable of distinguishing between diseased placenta [IUGR (Durhan et al., 2017; Saw et al., 2018a; Eroglu et al., 2020) and PE (Cimsit et al., 2015b)] from the normal placenta.

Acoustic Radiation Force Impulse (ARFI) excites short duration (0.1–0.5 ms) of high-intensity acoustic pulse on a single focus location to displace the tissue with a displacement of ~(10–20 μm) along the tissue direction, or perpendicular to the surface. The propagation shear wave speed (SWS) travel perpendicular to the plane of excitation, *c*_*s*_, is measured, which is a direct representative of tissue stiffness. In ARFI, SWS or Young Modulus is reported as a direct representation of tissue stiffness. Young Modulus can be converted from SWS using Equation 10 assuming homogenous, isotropic and elastic tissue. In ARFI, the higher propagation speed of waves indicates stiffer tissues.

(12) $${\text{c}}_{\text{s}}=\;\sqrt{\frac{\text{E}}{3\rho }}$$

where *c*_*s*_ is shear wave speed, *E* is Young Modulus and ρ is tissue density.

The mean of the *in vivo* SWS reported in multiple studies for normal, IUGR and PE placenta were ~0.99 m/s (0.71–2.21 m/s), 1.28 m/s (0.89–1.67 m/s), and 1.62 m/s (1.12–2.55 m/s), respectively (Cimsit et al., 2015a; Ohmaru et al., 2015; Alan et al., 2016a,b; Erbil et al., 2016; Wu et al., 2016). Although a significant difference was observed between normal and IUGR (Ohmaru et al., 2015), the range of SWS of the normal placenta was very large: SWS in the diseased placenta was overlapping with the SWS in the normal placenta. Also, readings from the *ex vivo* experiment were generally higher than those performed in the *in vivo* condition (Table 1).

Shear Wave Elastography (SWE) is similar to ARFI, but instead of focusing the pulse on a single focus location, it focuses on multiple focal points in rapid succession, generating a cylindrical shape shear wave that extended over a greater depth. Similar to ARFI, SWE measures the propagation wave speed travel perpendicular to the radiation pulse. Young Modulus can be converted from SWS using Equation 10. Similar results were also obtained in SWE where PE and IUGR placenta had significantly higher stiffness as compared to normal. SWE also suffered from high variability of measurements as shown in Kiliç et al. (2015) (Table 1).

Despite promising results, there remains much room to improve on elastography techniques to improve the ability to detect placenta diseases. Commercial elastography assumes linear, elastic, isotropic and incompressible material during stiffness computation, which violates conventional models that describe soft tissue as complex and heterogeneous materials. In fact, Varghese et al. study had shown that assuming a linear stress-strain relationship in elastography could result in misleading diagnosis at large applied compression, whereby the hard tissue can be misdiagnosed as soft tissue (Varghese et al., 2000). Further, soft tissue exhibits non-linear stress-strain relationship whereby its stiffness changes with strain or stress (Manoogian et al., 2009) and soft tissues also exhibits both viscous and elastic properties whereby its properties change with times, especially in SWE where the SWS will be affected by the excitation frequency. In order to obtain a more precise and accurate measurement from ultrasound elastography, non-linear stress-strain relationship and its viscoelasticity properties should be adopted in the algorithm during stiffness computation. Such efforts have been made in tumors (Wang and Insana, 2013) and breast cancer (Goenezen et al., 2012) elastography but not on the human placenta. Another noteworthy issue is that the placenta has been found to have substantial spatial heterogeneity in its mechanical properties (Saw et al., 2018b). This would thus require measurements to be performed over several locations of the placenta during evaluation.

## Future Research and Conclusion

Biomechanics studies such as those discussed above have improved our understanding of the umbilical-placenta system, and elastography, being an *in vivo* biomechanical measurement technique, showed promise as a detection technique for detection of pregnancy diseases. However, much more can be done improve our understanding of the umbilical-placenta system, and to lead to impact. Here, we suggest a few areas for such future work.

In terms of umbilical-placenta biofluid mechanics, one useful future endeavour will be to develop better models of flow and biotransport for the whole placenta vascular network, validated with clinical data, to study diseases such as PE and IUGR. Such models can be developed in a patient specific manner, where the model can be tuned to represent specific patients by matching ultrasound measurements, such as those of umbilical vascular flow velocities and vascular sizes. Such models may be useful in predicting the state of hypoxia during placenta insufficiency diseases, and may be able to help determine the best timing for early-delivery should it become necessary, which is important to outcomes. Successful modeling of placenta vacsular network flow during twin-to-twin transfusion may also be useful to estimating the hemodynamic situation, and to aid planning or laser surgery interventions.

Further, the biomechancial environment of the umbilical-placenta blood vessels are likely to play an important role in influencing their growth and remodeling, but we do not yet have complete information on such biomechanical environments, and do not yet understand their exact effects on growth. A combination of clinical and *in vivo* measurements and computational modeling of the flow environments will be valuable to address this gap, and may lead to better understanding of placenta insufficiency diseases, or even inspire treatment future approaches.

In terms of tissue mechanics of the placenta, we propose that continued evaluation of the placenta mechanical properties is important. A gap in the literature on this aspect is the essential difference of placenta mechanical behaviour between the *in vivo* and *ex vivo* conditions. To date, most placenta mechanical testing are based on post-delivery samples, but such samples have an essential difference from their *in vivo* condition. *In vivo*, the placenta is filled with blood, which exerts pressure and stresses on placenta tissues, conceivably providing turgidity to the organ, but post-delivery, the placenta are no longer filled with blood, and are flaccid. We suggest that controlled experiments to understand the differences and careful *in vivo* measurements of placenta mechanical behavior are important future work. This factor is also complicating direct validation of elastography results, preventing valid comparisons of *ex vivo* testing data to in elastography *in vivo* measurements.

Another useful future work is finite element modelling of impact of adverse events on the placenta, such as car crashes, blunt forces, and explosions, so as to understand their damage, and so as to help design protection devices, such as car air bags. Finite element modelling can also be done to model the elastography process, so as to derive more accurate ways to compute stiffness results from measurements. An example of where this will be useful is during strain elastography. In strain elastography, the palpation of the placenta is a complex biomechanical process, because the palpation is not resisted via compression by any structure on the amniotic fluid side, but is supported by adjacent placenta tissues via tension, conceivably leading to a complex deformation and stress field in the region of palpation. This process is not well-understood, and performing finite element modeling can improve our understanding of it.

In terms of elastography, although its promise in non-invasive determination of placenta mechanical properties for diagnosis purposes is well-known, its major limitations are its limited measurement accruacy and precision. In addressing this limitation, a few strategies may be useful. Firstly, more realistic mechanical properties can be incorporated into elastography. Currently, simplified models are used, such as using the Young's modulus instead of hyperelastic models, and using simplified viscoelasticity models. More advanced models may improve elastography's performance. A recent study proposed a novel algorithm to estimate 3D displacement and Young Modulus in elastography using phatom data (Hashemi et al., 2020). Secondly, with the growing sophistication of machine learning techniques, machine learning seems to be a promising approach to improve elastography performance. However, to achieve this, large elstography datasets with a valid validation approach are necessary. Thirdly, standardization approachs, such as robotic control of the transducer during elstography, and having an well-controlled stiffness reference during strain elastography may be helpful, and should be tested.

As for the umbilical cord, we suggest that biomechanical investigations can be helpful to understand its growth and development in health and during disease. The umbilical cord's development is likely responsive to the mechanical environment it experiences. For example its length and number of vascular coils per length are thought to be a result of the tugging tension and turning motions of the fetus. Biomechanical investigation may lead to an understanding of abnormally formed umbilical, such as excessively long cords, which tend to be associated with cord prolapse, torsion, knots, and entangement, and which are in turns, associated with fetal distress and anomalies. Computational modeling of the biotransport environment of fetuses during such complications may also be useful to understand the disease, and to determine whether intervention is warranted.

Finally, we conclude with our observation that biomechanical investigation of the umbilical-placenta system is under-explored but appear to have the potential to lead to novel discoveries and provide data to support clinical decisions.

## Data Availability Statement

The original contributions presented in the study are included in the article/supplementary material, further inquiries can be directed to the corresponding author/s.

## Author Contributions

All authors reviewed literature, participated in drafting, and critically revising the manuscript.

## Conflict of Interest

The authors declare that the research was conducted in the absence of any commercial or financial relationships that could be construed as a potential conflict of interest.

## Publisher's Note

All claims expressed in this article are solely those of the authors and do not necessarily represent those of their affiliated organizations, or those of the publisher, the editors and the reviewers. Any product that may be evaluated in this article, or claim that may be made by its manufacturer, is not guaranteed or endorsed by the publisher.
